# Empowering and disempowering climate and experiences of psychological violence in artistic gymnastics

**DOI:** 10.1007/s12662-023-00886-7

**Published:** 2023-06-14

**Authors:** Teresa Greither, Jeannine Ohlert

**Affiliations:** 1 Faculty of Psychology and Educational Science, Department Psychology, Ludwig-Maximilian University Munich, Munich, Germany; 2 Child- and Youth Psychiatry and Psychotherapy, University Hospital Ulm, Ulm, Germany; 3 Institute of Psychology, German Sport University Cologne, Cologne, Germany; 4 The German Research Center for Elite Sport Cologne—momentum, Cologne, Germany

**Keywords:** Psychological violence in sport, Emotional maltreatment, Empowering coaching, Self-determination theory, Achievement goal theory

## Abstract

In light of the continuing debate about coach-perpetrated violence and the maltreatment of athletes in the elite sports context, empowering and ethical approaches to coaching need to be investigated and proposed as evidence-based effective alternatives. This study aims to investigate the associations between motivational coaching climates and athletes’ experiences of psychological violence, as well as their effects on well-being. Using an anonymous cross-sectional online survey, artistic gymnasts’ perceptions of empowering and disempowering coach-created motivational climates, experiences of psychological violence in sport, well-being, and depressive symptoms were recorded quantitatively. Results indicate that a more disempowering climate predicts psychological violence, while an empowering climate was not a significant predictor. Regarding mental health outcomes, an effect of psychological violence on depressive symptoms and well-being was found, but no effect was found for the disempowering climate. Explorative analysis suggested that psychological violence mediates the relationship between disempowering coaching and mental health outcomes. Based on these findings, coach education should focus on prevention of psychological violence and minimization of disempowering climates. Further research should investigate the relations and effects of psychological violence and coaching climates more thoroughly, including the role of an empowering climate as a potential beneficiary alternative coaching method.

Artistic gymnastics has, in recent years, been at the heart of worldwide scandals surrounding allegations of psychological violence (e.g., Evans, Alesia, & Kwiatkowski, 2016). Reports of athletes, coaches, and scholars have emerged that demand a healthy way to success (e.g., Kerr, 2021). However, it is unclear what kind of coach education or coaching environments would be effective or helpful in reducing experiences that constitute psychological violence, specifically in the sports context. For effective research and intervention in the field, it is necessary to have a clear understanding of which behaviors are damaging to athletes and the ones that are more likely to protect athletes from harm. The concept of empowering coaching has been proposed by Appleton and Duda (Appleton, Ntoumanis, Quested, Viladrich, & Duda, 2016; Duda, 2013) as a theoretically sound and evidence-based framework for coach training programs. Its main goal is to foster a healthy social and psychological environment that optimizes athletes’ reasons for engagement, enjoyment, and commitment to sports through motivational processes. Various negative and positive associations have been researched within this framework, but it has so far not been brought into context with interpersonal violence in the sports context. Therefore, the aim of the current study was to explore the link between an empowering coaching style and experiences of interpersonal violence, especially in gymnasts.

## Psychological violence in the sports context

Stirling and Kerr (2008) characterized emotional abuse as patterns of harmful non-contact behaviors and behaviors that deny attention and support. These may occur within a critical relationship and can potentially be harmful regarding the victim’s affective, behavioral, cognitive, or physical well-being. The following categories of behaviors, which are taken from Parent et al. (2019) and Fortier, Parent, and Lessard (2020), will be referred to as psychological violence in this study: 1) verbal abuse and depreciation towards an athlete, e.g., shouting or belittling; 2) insufficient support or affection for an athlete, e.g., ignoring an athlete; 3) terrorizing or threatening an athlete, e.g., threatening to harm an athlete; 4) isolating or confining an athlete, e.g., limiting social contacts; 5) behaviors that promote corruption, exploitation, and adoption of destructive, antisocial, or unhealthy habits, e.g., supporting an athletes’ disordered eating habits to achieve a certain weight; 6) physical neglect, e.g., allowing (or encouraging) an athlete to train or compete with an injury against medical advice; 7) emotional neglect, e.g., refusing to provide psychological care to an athlete who clearly needs it; and 8) educational neglect, e.g., asking an athlete to drop out of school in order to practice sport.

Prevalence reports (Alexander, Stafford, & Lewis, 2011; Hartill et al., 2021; Ohlert, Vertommen, Rulofs, Rau, & Allroggen, 2021b; Vertommen et al., 2016; Willson, Kerr, Stirling, & Buono, 2021) have revealed that a large proportion of athletes have experienced behaviors that constitute psychological violence within the sports context. However, due to a lack of consensus in definition and methodology, comparisons can be difficult as numbers vary between studies (Ohlert, Schäfer, Rau, & Allroggen, 2021a). In the UK, it was reported that 75% of respondents had experienced at least one type of emotional harm while participating in organized sports (Alexander et al., 2011). For the Netherlands and Belgium, lower incidences were found, with 38% of respondents indicating to have experienced some form of psychological violence in the sports context (Vertommen et al., 2016). In a sample of elite athletes from Germany using a similar methodology to the study from Belgium and the Netherlands, Ohlert et al. (2021a) found much higher prevalence rates, with 87% of the examined German athletes reporting at least one incident of psychological violence. A total of 20% of the German sample reported behaviors that were classified as severe psychological violence, which is higher than the 10% reported for Belgian and Dutch athletes. Another recent prevalence report emerged from Canada (Willson et al., 2021), in which current and former national team members were surveyed. Emotional forms of harm were reported by 59% of current and 62% of retired athletes. Similarly, in Hartill et al.’s (2021) recent prevalence report of a large sample from six European countries (*N* = 10,302), 65% reported experiences of psychological violence within the sports context.

In addition, it has become clear that perpetrators, circumstances, and risk factors for abuse vary. Research has mostly covered coaches as perpetrators due to the often powerful influence they have over the careers and experiences of their athletes. The power relations between athletes and coaches can be described as asymmetric and may in consequence leave athletes more vulnerable to abuse (Mountjoy et al., 2016; Pinheiro, Pimenta, Resende, & Malcolm, 2014). In qualitative studies, it has been demonstrated that coaches may gain significant control over diverse areas of athletes’ lives, e.g., nutrition, recovery, education, or social relationships (Jacobs, Smits, & Knoppers, 2017; Pinheiro et al., 2014). In elite sports, immense trust is put into the coach by athletes and parents alike, which, especially when combined with a lack of parental supervision (e.g., when parents’ attendance is prohibited), constitutes a risk factor as well (Smits, Jacobs, & Knoppers, 2017; Stirling & Kerr, 2009). On a sociocultural level, psychological abuse is often accepted or normalized by coaches, parents, athletes, and even managers or authorities responsible for coaches’ conduct, as they view this kind of treatment as necessary to achieve success (Jacobs et al., 2017; Pinheiro et al., 2014; Stirling & Kerr, 2013). For the purpose of motivation, coaches may, for example, use intimidation, humiliation, or rejection to subject their athletes to physical and emotional challenges under the pretense of developing their mental strength (Owusu-Sekyere & Gervis, 2016). Parents and coaches might have experienced abuse in the athletic environment themselves, possibly contributing to the normalization, acceptance, and perpetuation of psychological violence, especially if these experiences remain unreflected (McMahon, Zehntner, McGannon, & Lang, 2020; Yabe et al., 2019).

Further, respecting hierarchies and obeying coaches’ orders are often desired characteristics and behaviors, but this puts athletes, especially child athletes, in a position of dependency and reduced power, which leaves them more vulnerable to subpar treatment (Jacobs et al., 2017; Smits et al., 2017; Stirling & Kerr, 2009). Indeed, Fournier, Parent, and Paradis (2021) could show that overadherence to certain sports ethics norms, especially the norm for self-sacrifice (Hughes & Coakley, 1991), increases athletes’ risk of experiencing psychological abuse from the coach. When looking at types of sports, aesthetic sports such as artistic gymnastics or figure skating seem to have a significantly higher prevalence rate of psychological violence than all other types of sports (Ohlert et al., 2021a).

The consequences of psychological violence in sports have mainly been investigated by qualitative studies. Elite athletes have reported several adverse effects connected with endured psychological abuse, e.g., anger, low mood, low self-efficacy, low self-esteem, body image issues, and anxiety. Others reported low motivation, less enjoyment of their sport, difficulties acquiring new skills, and lack of concentration (Stirling & Kerr, 2013). Another qualitative study by Kerr, Willson, and Stirling (2020) reported continuously evolving effects of psychological violence as athletes transition out of their active careers. Initial normalization of maltreatment shifts to feelings of compromised well-being and difficulties in daily life, and cumulates in long-term effects resembling symptoms of posttraumatic stress disorder such as lingering thoughts, flashbacks, and physical reactions (Kerr et al., 2020; Litvin, Kaminski, & Riggs, 2017).

Quantitative analyses of the consequences of interpersonal violence in sports are rather scarce. A study by Vertommen, Kampen, Schipper-van Veldhoven, Uzieblo, & Van Den Eede (2018) revealed that severe psychological violence is significantly associated with higher adult psychological distress and a lower quality of life. Those experiencing more or more severe violence also reportedly suffered from worse outcomes and mental health problems (Vertommen et al., 2018), which is in line with the extensive research available on child maltreatment in general (e.g., Kessler et al., 2010). Similarly to Vertommen et al. (2018), Ohlert, Rau, and Allroggen (2019) report that athletes who had experienced psychological violence had significantly lower quality of life and a higher risk for depression at the time of the survey in comparison to athletes who had not experienced psychological violence.

## Motivational coaching climates

The empowering and disempowering coach-created climates were developed by Duda and Appleton (Duda, 2013; Duda & Appleton, 2016a) to capture and describe the social psychological environment coaches create for their athletes, influencing their sporting experience. The concept is theoretically based on the self-determination theory (SDT, Deci & Ryan, 1985; Deci & Ryan, 2000) and the achievement goal theory (AGT, Ames, 1992; Nicholls, 1989, p. 19).

Achievement Goal Theory provides two perspectives on how the judgment of competence occurs: via the adoption of task- or ego-involving criteria. Task-involving criteria, e.g., the exertion of effort, improvement, learning, task mastery, or witnessing a personal best, are used in the task-goal perspective to define or judge success and competence (Ames, 1992; Nicholls, 1989). When coaches value task-involving criteria, they focus on every athlete working hard, doing their best, improving upon themselves, and getting better with their skills. On the other hand, if ego-involving criteria are emphasized, much focus is placed on performance comparisons with others and demonstrations of superiority. Ego-involving coaching behaviors, e.g., focusing on high-performing athletes, punishing mistakes, and fostering competition and comparisons among teammates, lead athletes to more ego-oriented goals, which means that they are more likely to compare their performance with others and develop a fear of failure (Appleton & Duda, 2016; Duda, 2001; Duda, 2013; Duda, Appleton, Stebbings, & Balaguer, 2017).

According to SDT (Deci & Ryan, 1985; Deci & Ryan, 2000), the strive for satisfaction of the three innate psychological needs for competence, autonomy, and relatedness is the fundamental motivational mechanism that drives peoples’ actions and behaviors. The experience of competence is defined as feeling effective in meeting demands of the environment, autonomy pertains to feelings of freedom and choice regarding an activity, and relatedness is described as feeling connected with others. Optimal functioning and autonomous motivation are likely to emerge if the needs for competence, autonomy, and relatedness are fulfilled to a greater extent.

Within the empowering and disempowering climates, aspects from both AGT and SDT are unified (Appleton et al., 2016; Appleton & Duda, 2016; Duda & Appleton, 2016b). The empowering climate dimension is made up of task-involving, autonomy-supportive, and socially supportive features. An empowering climate should lead to need satisfaction, autonomous motivation, a strong task-goal orientation, and, overall, to more positive experiences. In contrast, ego-involving and controlling coaching behaviors are categorized under the disempowering dimension, which is brought into connection with controlled motivation, ego-goal orientation, and comprised engagement in sports (Appleton & Duda, 2016; Duda, 2013). A key premise in Duda’s (2013) paradigm is that empowering and disempowering climates do not exist at opposite ends of a continuum. Coaches may create both empowering and disempowering environments by displaying behaviors that stem from both dimensions. For example, a coach might use a controlling behavior, such as offering rewards for good performances only and at the same time value lower-performing athletes for their contributions to the team—a socially supportive behavior. This highlights that empowering and disempowering dimensions can coexist and it is not a mistake or contradiction if coaches exhibit behaviors pertaining to both (Appleton & Duda, 2016). Several positive and negative effects have been investigated in relation to empowering and disempowering climates. An empowering climate and its subdimensions have been linked to, e.g., athletes’ increased enjoyment (Cheon, Reeve, Lee, & Lee, 2015; Jaakkola, Ntoumanis, & Liukkonen, 2016), overall higher self-worth (O’Rourke, Smith, Smoll, & Cumming, 2014; Quested & Duda, 2011), less burnout (Balaguer et al., 2012; Lemyre, Hall, & Roberts, 2008), emotions such as happiness and excitement (Ruiz, Appleton, Duda, Bortoli, & Robazza, 2021), and lower physical ill-being (Reinboth, Duda, & Ntoumanis, 2004). The subdimensions of the disempowering climate on the other hand (ego-involving and controlling coaching) have been brought into relation with the thwarting of basic psychological needs and ill-being in athletes. In the presence of need thwarting, athletes had a higher risk for disordered eating, symptoms of burnout, depression, negative affect, symptomatology, and disturbed physiological functioning (Bartholomew, Ntoumanis, Ryan, Bosch, & Thøgersen-Ntoumani, 2011). Appleton and Duda (2016) investigated effects of the empowering and disempowering climate on athletes’ health and functioning. Results indicate that both dimensions of the coaching climate interact regarding outcomes such as sport enjoyment, feelings of accomplishment, and physical symptoms. Into, Perttula, Aunola, Sorkkila, and Ryba (2019) investigated high school student athletes’ burnout symptoms in the sports and school context in association with an empowering and disempowering climate. Students with higher perceived empowering climate had fewer burnout symptoms in the sports environment compared to students with a higher perceived disempowering climate.

## Research question

Previous research suggests that both a negative coaching climate and experiences of psychological violence in sports are linked to negative consequences for athletes. For example, psychological need thwarting as well as psychological violence is linked to adverse outcomes such as higher risk for depression (Bartholomew et al., 2011; Vertommen et al., 2018). Furthermore, Ohlert, Schmitz, Schäfer-Pels, and Allroggen (2022) provided initial support that a coaching climate that is strongly empowering and minimal in disempowerment could be a valid approach to minimize the risk of sexual violence in sports. To date, however, no study has tried to investigate how experiences of psychological violence might relate to certain coaching styles or coaching climates, and how both aspects impact athletes’ well- and ill-being. From a theoretical point of view, it should be noted that using a disempowering climate does not mean using psychological violence as a means of coaching. Even though a disempowering climate consists of ego-involving and controlling coaching behaviors, these factors do not contain psychological violence in the theoretical definition. Therefore, it is possible to implement a highly disempowering climate without using elements of psychological violence. However, if a relationship between psychological violence and disempowering coaching exists, certain coaching styles should be added to the growing list of risk factors (or warning signs) for psychological violence.

Additionally, if empowering coaching is connected to less psychological violence, it supports the notion that these coaching behaviors should be a fundamental part of coach education, in an attempt to limit the use of damaging coaching methods. Accordingly, the aim of the current study was to investigate the connections between an empowering climate, experiences of psychological violence, and well-being, especially in artistic gymnasts, as they have been in the focus of public attention during the past years. In accordance with the theoretical considerations as well as previous studies, our expectations can be formulated as follows: 1) a higher disempowering climate should be associated with more experiences of psychological violence; 2) a higher empowering climate should be associated with fewer experiences of psychological violence; and 3) a higher disempowering climate and more psychological violence should both be associated with lower well-being in athletes.

During the research process, the question arose of whether effects of disempowering coaching on well-being and depressive symptoms might be mediated through experiences of psychological violence. It was hence decided to add an exploratory mediation analysis after preregistration: 4) psychological violence mediates the relationship between disempowering coaching and well-being and depressive symptoms.

## Methods

The Open Science Framework (OSF) was used to promote transparency through open science and to decrease incidences of questionable research practice in the current study. Hypotheses, methods, and data analytic approaches were published before accessing survey data. The preregistration can be found under this link: https://osf.io/wpav6. One additional non-preregistered exploratory hypothesis was added.

### Procedure

The study was conducted according to the ethical standards of the Declaration of Helsinki and the American Psychological Association (APA). Ethics approval by the Ethics Committee of the German Sports University Cologne was obtained. Due to the sensitivity of the research topic, participants had to be at least 16 years of age to be eligible for participation. The online survey was programmed with Unipark software (Unipark EFS Survey, 2020). The first author has good personal connections to the gymnastics community. For this reason, (former) athletes and gymnastics clubs were contacted directly to encourage participation in the survey. A snowball sampling effect was achieved, as some of the athletes shared the link via their personal social media sites. The survey was also reposted by several artistic gymnastics news websites. The participation period ended after 4 weeks.

Participants were informed about the study’s objective in a comprehensible way. A warning was included that some questions might be disconcerting as they refer to possibly unpleasant memories of their gymnastics career. If they felt the need to, affected persons were encouraged to seek help at a helpline and an organization for victims of abuse. A link to both was provided at the end of the survey. Gymnasts were instructed that participation was voluntary and anonymous. They were also made aware that they could terminate the survey at any time without consequences and were not required to answer all questions if they did not want to. Athletes were explicitly informed that data would be analyzed at the group level only to ensure anonymity.

### Participants

After cleaning the dataset and removing participants with incomplete questionnaires, data from 173 artistic gymnasts could be included in the analysis. The mean age of the participants was 27.01 years (standard deviation [SD] = 9.81 years). On average, they began their training in artistic gymnastics at the age of 5.83 years (SD = 2.09 years). 143 participants were of female and 27 of male gender. Three gymnasts did not indicate a gender. Out of the complete sample, 36 athletes defined themselves as currently active, 29 as currently inactive (e.g., due to the COVID-19 pandemic or injuries), and 98 as retired higher-level artistic gymnasts. About one quarter (28%) of the participants were (currently or previously) members of a regional or state selection team, 15% were members of the (extended) national team, and one third of the gymnasts (31%) indicated that they had never been members of any selection team.

### Measures

#### Psychological violence

To analyze experiences of psychological violence in sports, the psychological violence subscale of the Interpersonal Violence in Sports (IViS) questionnaire was administered. A German translation (IViS-D) has been established by Ohlert, Seidler, Rau, Rulofs, and Allroggen (2018). The IViS subscale for psychological violence encompasses 14 items on verbal behaviors, negative comments on body or performance, threats, and neglect. Four additional items were included that characterize psychological violence in sports according to the definition stated for this study: One item of the physical violence subscale of the IViS questionnaire was included as psychological violence, as it specifies a non-contact behavior with physical effects on the athlete. Three more items that appeared in the psychological violence section of both a Canadian survey (Willson et al., 2021) and the Violence Towards Athletes Questionnaire (VTAQ; Parent et al., 2019) were added as well to represent an exhaustive and up-to-date representation of experiences of psychological violence that may occur in sports.

Participants were asked to indicate if and how often they had experienced each situation from their current (or in the case of retired athletes, former) main coach during their sports career. The scale includes four answer options for each situation: “never,” “once,” “a few times,” and “regularly.”

#### Empowering and disempowering climates

To measure the empowering and disempowering climate, the Empowering and Disempowering Motivational Climate Questionnaire—Coach (EDMCQ‑C, 34 items) was applied (Appleton et al., 2016). A validated German version of the EDMCQ‑C was used for this study (Ohlert, 2018). The instrument consists of 34 items that can be divided into the empowering and disempowering climate dimensions. Again, participants were instructed to refer their answers to their current (or in the case of retired athletes, former) main coach. Answer options were presented on a five-point Likert scale ranging from “I do not agree at all” to “I completely agree.” Internal reliability was assessed in two different samples by Appleton et al. (2016), with Cronbach’s alphas ranging from 0.87 to 0.90 for the empowering dimension and 0.86 to 0.87 for the disempowering dimension.

#### Well-being

Well-being of the athletes was measured via two different questionnaires: the WHO-Five Well-Being Index (WHO5; Blom, Bech, Högberg, Larsson, & Serlachius, 2012; Bonsignore, Barkow, Jessen, & Heun, 2001; World Health Organization, 1998) and the Patient Health Questionnaire 2 (PHQ2; Kroenke, Spitzer, & Williams, 2003; Löwe, Kroenke, & Gräfe, 2005). The WHO‑5 is a well-known mental health questionnaire available in more than 30 languages. It assesses subjective psychological well-being on a scale from 0 to 5 (with options being “at no time,” “some of the time,” “less than half of the time,” “more than half of the time,” “most of the time,” or “all of the time”). Participants rate five positively worded statements concerning their well-being during the last 2 weeks (e.g., “I have felt calm and relaxed”). Points are added up to produce a sum score with a maximum of 25 points, which indicates an optimal well-being score. Internal consistency has been reported as good to excellent in a number of studies that assessed different versions of the WHO‑5, e.g., for adolescents Cronbach’s α = 0.82 was reported by de Wit, Pouwer, Gemke, Delemarre-van de Waal, and Snoek (2007) and for adults Cronbach’s α = 0.92 was reported by Brähler, Mühlan, Albani, and Schmidt (2007).

The PHQ‑2 is a short screening tool for depressive symptoms. The occurrence of two situations (“little interest or pleasure in doing things” and “feeling down, depressed, and hopeless”) is rated regarding the last 2 weeks on a scale from 0 to 3 (“not at all,” “several days,” “more than half the days,” and “nearly every day”). Answers are then added up for a sum scale ranging from 0 to 6. According to Kroenke et al. (2003), to screen for symptoms of major depression, a cut-off score of ≥ 3 is adequate as it detects depression in adults with 87% sensitivity and 78% specificity. Löwe et al. (2005) reported the internal consistency for an adult population, with Cronbach’s α = 0.83.

#### Demographics

Participating (former) athletes were asked to indicate their age and current status as a gymnast. Additionally, participants were asked to share the age at which they began with the sport of artistic gymnastics, their gender, and their highest achieved selection team membership (if applicable).

### Statistical analysis

All survey data were imported into and analyzed with the software R (R Core Team, 2020). In accordance with Vertommen et al. (2016) psychological violence items of the IViS‑D were recoded into mild, moderate, or severe incidences, thereby taking both type of situation and frequency of the violent behavior into account. Sum scores were obtained for the items of the IViS‑D, WHO‑5, and PHQ‑2. Higher scores in the IViS‑D indicate more or more severe experiences of psychological violence, while higher scores on the WHO‑5 refer to a higher well-being and higher PHQ‑2 scores to a higher risk for depression. For the EDMCQ‑C, mean scores for all subscales and the overarching dimensions (empowering and disempowering climates) were obtained for statistical analyses. Higher scores indicate that a climate dimension is pronounced more strongly.

Multiple regression analyses were used for hypothesis testing. For hypotheses one and two, disempowering and empowering climates (independent variables) and experiences of psychological violence (dependent variable) were entered into a regression model. For the impact on well-being, PHQ‑2 and WHO‑5 were entered into two separate regression models as dependent variables, with disempowering climates and psychological violence as independent variables.

For each regression model, tests for prerequisites were obtained. A variance inflation factor (VIF) below 5 was used as a threshold for multicollinearity (James, Witten, Hastie, & Tibshirani, 2013). Normal distribution of residuals was checked graphically, homoskedasticity via Breusch–Pagan Test using the lMTest package (Zeileis & Hothorn, 2002), and a graphic outlier analysis was undertaken using Cook’s distances. If the Breusch–Pagan test indicated heteroskedasticity, a heteroskedasticity-consistent covariance matrix was obtained by using the vcovHC() function of the sandwich package (Zeileis, 2004; Zeileis, Köll, & Graham, 2020) in combination with the lMTest package (Zeileis & Hothorn, 2002). As an exploratory hypothesis, a mediation was calculated to determine whether an effect of a disempowering coaching climate on the PHQ‑2 and the WHO‑5 might be mediated through experiences of psychological violence. The mediation was carried out using the psych package (Revelle, 2021).

## Results

### Initial data analysis

The scale reliability of the measures used ranged from acceptable to excellent. Means, standard deviations, reliability (Cronbachs α) of the questionnaire scales, and their intercorrelations are outlined in Table 1. Of the 173 participants, 93.1% (*n* = 161) reported at least one experience of psychological violence from their coach. 16.2% (*n* = 28) reported only violence categorized as mild, 34.1% (*n* = 59) reported at least one situation that was categorized as moderate, and 42.8% (*n* = 74) indicated at least one experience of psychological violence that was categorized as severe. The remaining 6.9% (*n* = 12) of participants did not report psychological violence from their coach. Compared to other samples (Belz, Kleinert, Ohlert, Rau, & Allroggen, 2018; Brähler et al., 2007; Ohlert et al., 2021b), participants’ well-being as measured by the WHO‑5 was mediocre to low, while the depression screen (PHQ-2) showed relatively high scores. Coaching climates of the sample can be characterized as moderately empowering and relatively high in terms of disempowering (Appleton & Duda, 2016).

**Table 1:** Reliability, means, standard deviation, and intercorrelation of questionnaire scales

Variable	*n*	α	M	SD	1	2	3	4
1. Psy. violence	173	0.92	12.62	10.67	–	–	–	–
2. Empowering	173	0.96	3.48	0.95	−0.73**	–	–	–
3. Disempowering	173	0.91	2.90	0.86	0.83**	−0.84**	–	–
4. WHO‑5	167	0.82	14.71	4.62	−0.46**	0.46**	−0.42**	–
5. PHQ‑2	169	0.78	1.85	1.46	0.45**	−0.38**	0.38**	−0.62**

*Psy. violence* psychological violence, *Empowering* Empowering Climate, *Disempowering* Disempowering Climate, *WHO-5* World Health Organisation Five Well-Being Index, *PHQ-2* Patient Health Questionnaire Two, *M* mean, *SD* standard deviation

*p < 0.05, **p < 0.01

### Hypotheses testing

The first two hypotheses were tested using multiple regression with disempowering and empowering coaching as predictors of experiences of psychological violence. A significant model was revealed, which explained 68.9% of the variance of psychological violence. The disempowering climate emerged as a significant predictor for psychological violence (*b* = 9.00, *p* = < 0.001), while the empowering climate was not a significant predictor (*b* = −1.41, *p* = 0.24; see Table 2).

**Table 2:** Hypotheses 1 and 2: multiple regression with psychological violence as the dependent variable

Independent variable	*b*	SE	95% CI		*t*	*p*-value
			LL	UL		
Empowering	−1.41	1.19	−3.13	0.30	−1.19	0.237
Disempowering	9.00	1.28	7.10	10.90	7.01	< 0.001
Constant	−8.56	7.62	−19.60	2.48	−1.19	0.263
Observations	173					
*R*^*2*^	0.69					
Adjusted *R*^*2*^	0.69					
*F*-statistics	191.40 (*df* = 2; 170); *p* < 0.001					

*Empowering* Empowering Climate, *Disempowering* Disempowering Climate, *b* unstandardized regression coefficient, *SE* standard error, *CI* confidence interval, *LL* lower limit, *UL* upper limit

For hypothesis three, two multiple regression models with psychological violence and disempowering climate as predictors, and WHO‑5 and PHQ‑2 as criteria, were computed. For WHO‑5, a significant model was revealed which explained 21% of the variance of WHO‑5. Only psychological violence emerged as a significant predictor (*b* = −0.16, *p* = < 0.01), while the disempowering climate was not significant (*b* = −0.61, *p* = 0.40). Detailed results can be viewed in Table 3.

**Table 3:** Hypothesis 3: multiple regression with WHO‑5 as the dependent variable

Independent variables	*b*	SE	95% CI		*t*	*p*-value
			LL	UL		
Psychological violence	−0.16	0.06	−0.27	−0.05	−2.81	0.003
Disempowering	−0.61	0.73	−1.94	0.72	−0.83	0.368
Constant	18.52	1.53	15.63	21.40	12.07	< 0.001
Observations	167					
*R*^*2*^	0.22					
Adjusted *R*^*2*^	0.21					
*F*-statistics	22.78 (*df* = 2; 164); *p* < 0.001					

*Disempowering* Disempowering Climate, *b* unstandardized regression coefficient, *SE* standard error, *CI* confidence interval, *LL* lower limit, *UL* upper limit

For the PHQ‑2, a significant model resulted which explained 20% of the variance of the PHQ‑2. Psychological violence again emerged as a significant predictor (*b* = −0.06, *p* = < 0.001), while the disempowering climate was a non-significant predictor (*b* = −0.05, *p* = 0.810). Detailed results can be viewed in Table 4.

**Table 4:** Hypothesis 3: multiple regression with PHQ‑2 as the dependent variable

Independent variables	*b*	SE	95% CI		*t*	*p*-value
			LL	UL		
Psychological violence	0.06	0.02	0.03	0.09	3.47	< 0.001
Disempowering	0.05	0.21	−0.36	0.47	0.25	0.810
Constant	0.96	0.46	0.04	1.87	2.01	0.040
Observations	169					
*R*^*2*^	0.21					
Adjusted *R*^*2*^	0.20					
*F*-statistics	21.51 (*df* = 2; 166), *p* < 0.001					

*Disempowering* Disempowering Climate, *b* unstandardized regression coefficient, *SE* standard error, *CI* confidence interval, *LL* lower limit, *UL* upper limit

**Fig. 1:**
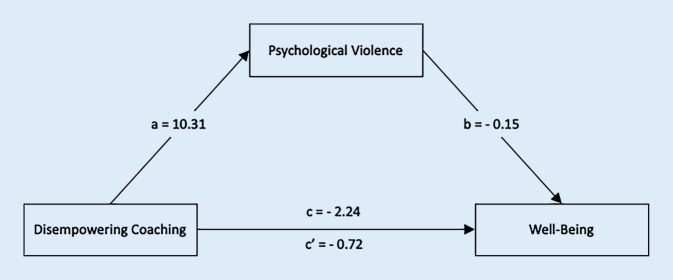
Mediation model for (exploratory) hypothesis 4. *c* Total effect of Disempowering Coaching on Well-Being, *c’* Direct effect of Disempowering Coaching on Well-Being, *a* Effect of Disempowering Coaching on Psychological Violence, *b* Effect of Psychological Violence on Well-Being

Analysis for exploratory hypothesis four demonstrated that the relationship between disempowering coaching and well-being as well as depressive symptoms was mediated by psychological violence. As can be seen in Fig. 1, the total effect (c) of a disempowering coaching climate on well-being was −2.24 (Standard Error SE = 0.37, *t* = −5.99, *df* = 171, *p* < 0.001). The direct effect (c’) of a disempowering coaching climate on well-being removing psychological violence was −0.72 (SE = 0.66, *t* = −1.09, *df* = 170, *p* = 0.28). The indirect effect (ab) was −1.52. Unstandardized indirect effects were computed for 5000 samples, and 95% confidence intervals (CI) were obtained. The mean bootstrapped indirect effect was −1.54 (SE = 0.59), with CI ranging from −2.71 to −0.41 and thus statistically significant.

**Fig. 2:**
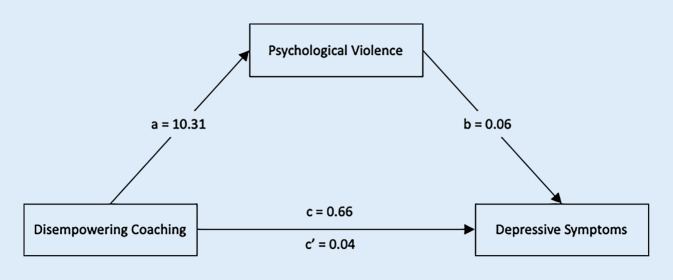
Mediation model for (exploratory) hypothesis 4

The exploratory analysis for depressive symptoms revealed a significant mediation effect as well. The mediation model is depicted in Fig. 2. The total effect (c) of disempowering coaching climates on psychological violence was 0.66 (SE = 0.12, *t* = 5.45, *df* = 171, *p* < 0.001). The direct effect of disempowering coaching climates on depressive symptoms became insignificant when removing psychological violence, with c’ = 0.04 (SE = 0.21, *t* = 0.2, *df* = 170, *p* = 0.84). A significant indirect effect (ab) was obtained: The mean bootstrapped indirect effect with 5000 samples was = 0.61 (SE = 0.19, lower CI = 0.24, upper CI = 0.97).

## Discussion

This study’s objective was to investigate associations between psychological violence from the coach, empowering and disempowering motivational coaching climates, and athletes’ well-being in a sample of artistic gymnasts. Results showed that a disempowering climate was a significant predictor of psychological violence perpetrated by the coach. In contrast, an empowering climate did not significantly predict psychological violence when the disempowering climate was controlled for. Additionally, psychological violence significantly predicted well-being and depressive symptoms, while the disempowering climate did not. Explorative hypothesis testing revealed a significant mediation effect of psychological violence between disempowering coaching and well-being as well as depressive symptoms.

Our study gives first insights into the connection between the coaching climate and psychological violence and indicates that a robust association between a disempowering climate and psychological violence exists, as a large effect size occurred in this case. Athletes experiencing a motivational climate that is highly disempowering, i.e., highly controlling and ego involving, are more likely to experience more psychological violence from their coach. Consequently, a motivational climate that is less disempowering is associated with fewer incidences of psychological violence from the coach. This finding is in line with research that found a highly disempowering climate and its subdimensions to be linked to worse experiences and health outcomes for athletes (Bartholomew, Ntoumanis, & Thøgersen-Ntoumani, 2010; Ruiz et al., 2021; Stebbings, Taylor, & Spray, 2015).

A more empowering climate did not significantly predict less psychological violence in the regression analysis, which was not an expected result. The empowering climate is based on autonomy support, social support, and a healthy task-goal perspective. It has been associated with positive outcomes for athletes (Appleton et al., 2016; Appleton & Duda, 2016). It was in consequence hypothesized that a more empowering climate would predict fewer adverse experiences for athletes. Based on this result, it could be concluded that it might be the extent of the disempowering climate that is predictive of abuse, rather than the presence or absence of a more- or less-empowering climate. However, according to Duda and Appleton’s conceptualization, the empowering and disempowering climates are not mutually exclusive and may actually be present simultaneously (Appleton et al., 2016; Duda, 2013). Therefore, an explanation for this finding might be that a low-empowering climate, while probably not ideal, is not necessarily highly disempowering and thus not associated with psychological violence. By implication, if a highly empowering climate is on display, the absence of disempowering strategies is not guaranteed, and athletes might still be at risk of experiencing psychological violence from their coach.

When turning to the results concerning well-being and depressive symptoms, only psychological violence emerged as a significant predictor, although it was hypothesized that a higher disempowering climate would predict these health outcomes as well. One explanation for this finding might be that experiences of psychological violence had an overall larger effect on athletes’ mental health, while the influence of the disempowering climate diminished in comparison. Another explanation for this result emerges when examining the operationalization of the disempowering coaching behaviors within the EDMCQ questionnaire. Some items in the questionnaire appear to resemble behaviors that might constitute psychological violence depending on the context in which they occur and the extent to which they are executed. For example, shouting at athletes or punishing them for mistakes are used as items of the disempowering climate dimension, but might at the same time constitute psychological violence, especially when extreme forms occur. Still, it should be noted that from a theoretical point of view, a disempowering climate does not necessarily include psychological violence, but can be enforced strongly without even using any kind of psychological violence. Thus, from a purely theoretical perspective, a disempowering climate should show only medium-sized correlations to psychological violence. However, given the operationalization of the items of a disempowering climate within the EDMCQ, some overlaps with psychological violence can be found and are presumably partly responsible for the high correlations between the two constructs. It hence seems conceivable that athletes exposed to such behaviors might report lower well-being and higher depressive symptoms mainly due to the psychological violence they experienced (as part of the disempowering climate).

In an explorative approach, psychological violence did indeed mediate the relationship between disempowering coaching and well-being as well as depressive symptoms. These findings can be taken as a first indicator that adverse effects or negative consequences of certain coaching styles might be better explained by experiences of psychological violence, but further research is warranted to establish a clear connection and should also take into account the possible overlaps of the two constructs at the level of operationalization. Subsequently, prospective studies examining the negative consequences of coach-created climates should either use more defined questionnaires or include questions about experiences of psychological violence, as controlling for such might be helpful to better understand which behaviors are detrimental to athletes’ health and well-being.

To assess the generalizability of the results, sample characteristics need to be discussed. First and foremost, the study population consisted of a convenience sample and might be biased, as the author partly contacted them. The majority of participants were females, so a gender bias needs to be assumed as well. The prevalence of psychological violence appears to be very high, with a rate of 93% in this sample, especially compared to more general reports from organized sports (e.g., 65% in Hartill et al., 2021). Nevertheless, this study’s prevalence rate is comparable to that of Ohlert et al.’s sample (2021a), where 95% of athletes in aesthetic sports (including artistic gymnastics) had been affected by psychological violence. Ohlert et al. (2021a) did not directly state the prevalence of severe psychological violence for aesthetic sports, but 21% of the overall sample (including all sports) had been affected, while in the current study, 43% of athletes reported at least one incident that was categorized as severe. An explanation for the higher severity of experiences might be that four more items were added to the IViS‑D scale that constituted psychological abuse according to this study’s definition, leading to a more exhaustive representation of psychological violence. Consequently, the sample seems to be in part representative for artistic gymnastics, even though overall experiences appear to be more severe. Replicating the study with a sample outside of aesthetic sports with a more representative sample and a balanced gender ratio would be helpful to assess the transferability of results to sports coaching in general. Another aspect to consider is that the perceived coaching climates were generally characterized as moderately empowering, with a mean of 3.48 (SD = 0.95). A mean value of 2.90 (SD = 0.86) was detected for the disempowering climate, which, while lower than the mean for the empowering dimension, still presents a considerably high level of disempowering climate. To corroborate the associations presented in this study, replication in a sports environment where the disempowering climate is lower and the empowering climate more strongly pronounced is advisable and might enhance insight into the possible protective role of a more empowering climate in relation to experiences of psychological violence. In addition, studies should disentangle the operationalizations of a disempowering climate from psychological violence, in order to examine the distinct effects of and connection between the two constructs.

The well-being of participants measured by the WHO‑5 was mediocre to low in comparison with a norm sample (Brähler et al., 2007) and other samples of elite athletes (Belz et al., 2018; Ohlert et al., 2021b). The depressive symptom screen PHQ‑2 revealed a mean which is also higher than the scores reported by Belz et al. (2018) and Ohlert et al. (2021b). During the survey phase of this research project, participants’ mental health and well-being might have been negatively influenced due to the ongoing impact of the COVID-19 pandemic, e.g., caused by lockdown measures or health concerns, therefore explaining the overall lower well-being. The effect of psychological violence on the indicators of depressive symptoms (*b* = −0.06, *p* = < 0.001) and well-being (*b* = −0.16, *p* = < 0.01) is small, yet significant and of practical relevance. Experiences of psychological violence in sports, which probably occurred much earlier, still emerged as a significant predictor of current depressive symptoms and compromised well-being. In line with Vertommen et al. (2018), this further corroborates the need for broader actions in child protection in organized sports. While not the main focus of this study, this finding provides additional evidence that psychological violence has long-lasting and far-reaching effects on athletes’ welfare. Another constraint that needs to be considered is that other forms of violence, e.g., sexual violence or physical violence, as well as any other kind of adverse experience outside of the sports context, might have influenced participants’ current well-being, but these effects were not accounted for here. Another flaw of the survey sample is that only participants above the age of 16 were allowed to take part due to ethical reasons. Most artistic gymnasts start (and often end) their careers much younger than 16, and many squad athletes are below this age threshold. Their experiences are not represented in this study. Furthermore, due to the cross-sectional data, no causality can be assumed between the coaching climates, experiences of psychological violence, and their effects on athlete welfare.

Despite its limitations, this study carries several implications for research, but also for coaching education. Our results give first insights into the fact that controlling and ego-involving coaching behaviors might precede or accompany incidences that constitute psychological violence and foster an environment that normalizes or facilitates such detrimental actions. Thus, when designing coach education programs, focusing more on how to reduce a disempowering climate could be helpful. Although a lower risk for psychological violence could not be predicted through higher levels of an empowering climate, this finding does not diminish the fact that the empowering climate and its subdimensions have been shown to hold several beneficial effects for athletes’ welfare and functioning (Appleton & Duda, 2016; Fenton, Duda, Quested, & Barrett, 2014). Beyond this, a recent study by Ohlert et al. (2022) has already produced preliminary evidence that a coaching climate that is highly empowering and low in disempowerment could be a useful approach to prevent sexual violence in sports. Since all forms of interpersonal violence are strongly interconnected, with psychological violence often used within a grooming process for more severe incidents (Ohlert et al., 2021a; Ohlert et al., 2021b), it seems likely that an evidence-based positive coaching climate such as the empowering climate might aid the prevention of all forms of interpersonal violence.

Regarding further research, this study corroborates the existing evidence that psychological violence is pervasive, at least in the sport of artistic gymnastics. More research should be undertaken to understand the root causes of this widespread problem. A better and more differentiated understanding of why coaches use damaging methods is essential to help design effective educational programs that specifically target the problem at hand. Putting these results in perspective, coaches are not the only perpetrators of violence against athletes, yet they influence their athletes’ behavior through the social environment they create.

Taking together the findings of the study, it seems necessary to clearly delineate subpar coaching practices like disempowering coaching dimensions from psychological violence, especially for sports practitioners such as coaches. If behaviors like shouting and yelling at athletes or ignoring them for poor performance are defined as a coaching style, it is implausible to characterize the same behaviors as psychological violence. Moreover, explaining the alternatives to these behaviors and how they positively impact athletes’ and even coaches’ well-being needs to be emphasized in coach education about psychological violence. The movement #gymnastalliance has created thorough awareness of the problem of interpersonal violence in sports, and this momentum should be used by researchers and sports practitioners alike to push for the implementation of coaching education that helps protect athletes and puts their well-being at the center.

## Declarations

The study was conducted in accordance with the Declaration of Helsinki and approved by the Ethics Committee of the German Sports University Cologne. Informed consent was obtained from all subjects involved in the study.

## References

[CR1] Alexander, K., Stafford, A., & Lewis, R. (2011). *The experiences of children participating in organized sport in the UK*. Study report. NSPCC Child Protection Research Centre.

[CR2] Ames, C. (1992). Achievement goals, motivational climate, and motivational processes. In G. C. Roberts (Ed.), *Motivation in sport and exercise* (pp. 161–176). Human Kinetics.

[CR3] Appleton, P. R., & Duda, J. (2016). Examining the interactive effects of coach-created empowering and disempowering climate dimensions on athletes’ health and functioning. *Psychology of Sport and Exercise*, *26*, 61–70. 10.1016/j.psychsport.2016.06.007.

[CR4] Appleton, P. R., Ntoumanis, N., Quested, E., Viladrich, C., & Duda, J. (2016). Initial validation of the coach-created empowering and disempowering motivational climate questionnaire (EDMCQ-C). *Psychology of Sport and Exercise*, *22*, 53–65. 10.1016/j.psychsport.2015.05.008.

[CR5] Balaguer, I., González, L., Fabra, P., Castillo, I., Mercé, J., & Duda, J. (2012). Coaches’ interpersonal style, basic psychological needs and the well-and ill-being of young soccer players: A longitudinal analysis. *Journal of Sports Sciences*. 10.1080/02640414.2012.731517. 23062028 10.1080/02640414.2012.731517

[CR7] Bartholomew, K., Ntoumanis, N., & Thøgersen-Ntoumani, C. (2010). The controlling interpersonal style in a coaching context: development and initial validation of a psychometric scale. *Journal of Sport & Exercise Psychology*, *32*, 193–216. 10.1123/jsep.32.2.193. 20479478 10.1123/jsep.32.2.193

[CR6] Bartholomew, K., Ntoumanis, N., Ryan, R. M., Bosch, J. A., & Thøgersen-Ntoumani, C. (2011). Self-determination theory and diminished functioning: The role of interpersonal control and psychological need thwarting. *Personality & Social Psychology Bulletin*, *37*(11), 1459–1473. 10.1177/0146167211413125. 21700794 10.1177/0146167211413125

[CR8] Belz, J., Kleinert, J., Ohlert, J., Rau, T., & Allroggen, M. (2018). Risk for depression and psychological well-being in German national and state team athletes—Associations with age, gender, and performance level. *Journal of Clinical Sport Psychology*, *12*(2), 160–178. 10.1123/jcsp.2016-0024.

[CR9] Blom, E. H., Bech, P., Högberg, G., Larsson, J. O., & Serlachius, E. (2012). Screening for depressed mood in an adolescent psychiatric context by brief self-assessment scales—Testing psychometric validity of WHO‑5 and BDI‑6 indices by latent trait analyses. *Health and Quality of Life Outcomes*, *10*(1), 149. 10.1186/1477-7525-10-149. 23227908 10.1186/1477-7525-10-149 PMC3575311

[CR10] Bonsignore, M., Barkow, K., Jessen, F., & Heun, R. (2001). Validity of the five-item WHO Well-Being Index (WHO-5) in an elderly population. *European Archives of Psychiatry and Clinical Neuroscience*, *251*(Suppl 2), 27–31. 10.1007/BF03035123. 10.1007/BF03035123 11824831

[CR11] Brähler, E., Mühlan, H., Albani, C., & Schmidt, S. (2007). Teststatistische Prüfung und Normierung der deutschen Versionen des EUROHIS-QOL Lebensqualität-Index und des WHO‑5 Wohlbefindens-Index. *Diagnostica*, *53*(2), 83–96. 10.1026/0012-1924.53.2.83.

[CR12] Cheon, S. H., Reeve, J., Lee, J., & Lee, Y. (2015). Giving and receiving autonomy support in a high-stakes sport context: A field-based experiment during the 2012 London Paralympic Games. *Psychology of Sport and Exercise*, *19*, 59–69. 10.1016/j.psychsport.2015.02.007.

[CR14] Deci, E. L., & Ryan, R. M. (1985). *Intrinsic motivation and self-determination in human behavior*. Springer US. 10.1007/978-1-4899-2271-7.

[CR15] Deci, E. L., & Ryan, R. M. (2000). The “what” and “why” of goal pursuits: human needs and the self-determination of behavior. *Psychological Inquiry*, *11*(4), 227–268. 10.1207/S15327965PLI1104_01.

[CR16] Duda, J. (2001). Goal perspectives research in sport: Pushing the boundaries and clarifying some misunderstandings. In G. C. Roberts (Ed.), *Advances in motivation in sport and exercise* (pp. 129–182). Human Kinetics.

[CR17] Duda, J. (2013). The conceptual and empirical foundations of Empowering Coaching^TM^: Setting the stage for the PAPA project. *International Journal of Sport and Exercise Psychology*, *11*(4), 311–318. 10.1080/1612197X.2013.839414.

[CR18] Duda, J., & Appleton, P. R. (2016a). Empowering and disempowering coaching climates: conceptualization, measurement considerations, and intervention implications. In M. Raab, P. Wylleman, R. Seiler, A.-M. Elbe & A. Hatzigeorgiadis (Eds.), *Sport and exercise psychology research* (pp. 373–388). Academic Press. 10.1016/B978-0-12-803634-1.00017-0.

[CR19] Duda, J., & Appleton, P. R. (2016b). Empowering and disempowering coaching climates: conceptualization, measurement considerations, and intervention implications. In M. Raab, P. Wylleman, R. Seiler, A.-M. Elbe & A. Hatzigeorgiadis (Eds.), *Sport and exercise psychology research* (pp. 373–388). Academic Press. 10.1016/B978-0-12-803634-1.00017-0.

[CR20] Duda, J., Appleton, P. R., Stebbings, J., & Balaguer, I. (2017). Towards more empowering and less disempowering environments in youth sport. In C. J. Knight, C. G. Harwood & D. Gould (Eds.), *Sport psychology for young athletes* (1st edn., pp. 81–93). Routledge. 10.4324/9781315545202-8.

[CR21] Evans, T., Alesia, M., & Kwiatkowski, M. Former. USA Gymnastics doctor accused of abuse. *IndyStar*. Retrieved on July 19, 2021, from https://www.indystar.com/story/news/2016/09/12/former-usa-gymnastics-doctor-accused-abuse/89995734/ (Created 12 Sept 2016). Accessed 19 Jul 2021

[CR22] Fenton, S. A. M., Duda, J., Quested, E., & Barrett, T. (2014). Coach autonomy support predicts autonomous motivation and daily moderate-to-vigorous physical activity and sedentary time in youth sport participants. *Psychology of Sport and Exercise*, *15*(5), 453–463. 10.1016/j.psychsport.2014.04.005.

[CR23] Fortier, K., Parent, S., & Lessard, G. (2020). Child maltreatment in sport: Smashing the wall of silence: a narrative review of physical, sexual, psychological abuses and neglect. *British Journal of Sports Medicine*, *54*(1), 4–7. 10.1136/bjsports-2018-100224. 31653778 10.1136/bjsports-2018-100224

[CR24] Fournier, C., Parent, S., & Paradis, H. (2021). The relationship between psychological violence by coaches and conformity of young athletes to the sport ethic norms. *European Journal for Sport and Society*. 10.1080/16138171.2021.1878436.

[CR25] Hartill, M., Rulofs, B., Lang, M., Vertommen, T., Allroggen, M., Cirera, E., Diketmueller, R., Kampen, J., Kohl, A., Martin, M., Nanu, I., Neeten, M., Sage, D., & Stativa, E. (2021). *CASES: child abuse in sport: European statistics—Project report*. Edge Hill University.

[CR26] Hughes, R., & Coakley, J. (1991). Positive deviance among athletes: the implications of over conformity to the sports ethic. *Sociology of Sport Journal*, *8*, 307–325. 10.1123/ssj.8.4.307.

[CR27] Into, S., Perttula, V.-M., Aunola, K., Sorkkila, M., & Ryba, T. (2019). Relationship between coaching climates and student-athletes’ symptoms of burnout in school and sports. *Sport, Exercise, and Performance Psychology*. 10.1037/spy0000180.

[CR28] Jaakkola, T., Ntoumanis, N., & Liukkonen, J. (2016). Motivational climate, goal orientation, perceived sport ability, and enjoyment within Finnish junior ice hockey players: Achievement goal theory and enjoyment. *Scandinavian Journal of Medicine & Science in Sports*, *26*(1), 109–115. 10.1111/sms.12410. 25648198 10.1111/sms.12410

[CR29] Jacobs, F., Smits, F., & Knoppers, A. (2017). “You don’t realize what you see!”: The institutional context of emotional abuse in elite youth sport. *Sport in Society*, *20*(1), 126–143. 10.1080/17430437.2015.1124567.

[CR30] James, G., Witten, D., Hastie, T., & Tibshirani, R. (2013). *An introduction to statistical learning: with applications in R*. Springer. 10.1007/978-1-4614-7138-7.

[CR31] Kerr, G. Next steps in the safe sport journey: from prevention of harm to optimizing experiences. *The sport information resource centre*. Retrieved on August 3, 2022 from https://sirc.ca/blog/next-steps-in-the-safe-sport-journey/ (Created 19 Apr 2021). Accessed 3 Aug 2022

[CR32] Kerr, G., Willson, E., & Stirling, A. (2020). “It was the worst time in my life”: The effects of emotionally abusive coaching on female Canadian national team athletes. *Women in Sport and Physical Activity Journal*, *28*(1), 81–89. 10.1123/wspaj.2019-0054.

[CR33] Kessler, R. C., McLaughlin, K. A., Green, J. G., Gruber, M. J., Sampson, N. A., Zaslavsky, A. M., Aguilar-Gaxiola, S., Alhamzawi, A. O., Alonso, J., Angermeyer, M., Benjet, C., Bromet, E., Chatterji, S., de Girolamo, G., Demyttenaere, K., Fayyad, J., Florescu, S., Gal, G., Gureje, O., et al. (2010). Childhood adversities and adult psychopathology in the WHO World Mental Health Surveys. *The British Journal of Psychiatry*, *197*(5), 378–385. 10.1192/bjp.bp.110.080499. 21037215 10.1192/bjp.bp.110.080499 PMC2966503

[CR34] Kroenke, K., Spitzer, R. L., & Williams, J. B. W. (2003). The patient health questionnaire-2: Validity of a two-item depression screener. *Medical Care*, *41*(11), 1284–1292. 10.1097/01.MLR.0000093487.78664.3C. 14583691 10.1097/01.MLR.0000093487.78664.3C

[CR35] Lemyre, P.-N., Hall, H. K., & Roberts, G. C. (2008). A social cognitive approach to burnout in elite athletes. *Scandinavian Journal of Medicine & Science in Sports*, *18*(2), 221–234. 10.1111/j.1600-0838.2007.00671.x. 17617173 10.1111/j.1600-0838.2007.00671.x

[CR36] Litvin, J. M., Kaminski, P. L., & Riggs, S. A. (2017). The complex trauma inventory: A self-report measure of posttraumatic stress disorder and complex posttraumatic stress disorder. *Journal of Traumatic Stress*, *30*(6), 602–613. 10.1002/jts.22231. 29160557 10.1002/jts.22231

[CR37] Löwe, B., Kroenke, K., & Gräfe, K. (2005). Detecting and monitoring depression with a two-item questionnaire (PHQ-2). *Journal of Psychosomatic Research*, *58*(2), 163–171. 10.1016/j.jpsychores.2004.09.006. 15820844 10.1016/j.jpsychores.2004.09.006

[CR38] McMahon, J., Zehntner, C., McGannon, K. R., & Lang, M. (2020). The fast-tracking of one elite athlete swimmer into a swimming coaching role: A practice contributing to the perpetuation and recycling of abuse in sport? *European Journal for Sport and Society*, *17*(3), 265–284. 10.1080/16138171.2020.1792076.

[CR39] Mountjoy, M., Brackenridge, C., Arrington, M., Blauwet, C., Carska-Sheppard, A., Fasting, K., Kirby, S., Leahy, T., Marks, S., Martin, K., Starr, K., Tiivas, A., & Budgett, R. (2016). International Olympic Committee consensus statement: Harassment and abuse (non-accidental violence) in sport. *British Journal of Sports Medicine*, *50*(17), 1019–1029. 10.1136/bjsports-2016-096121. 27118273 10.1136/bjsports-2016-096121

[CR40] Nicholls, J. G. (1989). *The competitive ethos and democratic education*. Harvard University Press.

[CR41] Ohlert, J. (2018). Erfassung des Empowerment Klimas in Sportgruppen – erste Validierung des Fragebogens zum Trainer*innen-induzierten Empowerment Klima (FTEK). In U. Borges, L. Bröker, S. Hoffmann, T. Hosang, S. Laborde, R. Liepelt, B. Lobinger, J. Löffler, L. Musculus & M. Raab (Eds.), *50. Jahrestagung der asp „Die Psychophysiologie der Handlung“* (p. 118). Deutsche Sporthochschule Köln.

[CR45] Ohlert, J., Seidler, C., Rau, T., Rulofs, B., & Allroggen, M. (2018). Sexual violence in organized sport in Germany. *German Journal of Exercise and Sport Research*, *48*(1), 59–68. 10.1007/s12662-017-0485-9.

[CR42] Ohlert, J., Rau, T., & Allroggen, M. (2019). Association between sexual violence experiences and well-being and risk for depression in elite athletes depends on the context of the incidents. *Journal of Clinical Sport Psychology*, *13*(2), 311–329. 10.1123/jcsp.2019-0008.

[CR43] Ohlert, J., Schäfer, A., Rau, T., & Allroggen, M. (2021a). Psychische Gewalt gegen Athletinnen und Athleten: Ein Problem nicht nur im Turnsport. *Leistungssport*, *51*(5), 14–19.

[CR46] Ohlert, J., Vertommen, T., Rulofs, B., Rau, T., & Allroggen, M. (2021b). Elite athletes’ experiences of interpersonal violence in organized sport in Germany, the Netherlands, and Belgium. *European Journal of Sport Science*, *21*(4), 604–613. 10.1080/17461391.2020.1781266. 32524909 10.1080/17461391.2020.1781266

[CR44] Ohlert, J., Schmitz, H., Schäfer-Pels, A., & Allroggen, M. (2022). An empowering climate as a protective factor against sexual violence in sport? *Social Sciences*, *11*(8), 330. 10.3390/socsci11080330.

[CR47] O’Rourke, D. J., Smith, R. E., Smoll, F. L., & Cumming, S. P. (2014). Relations of parent- and coach-initiated motivational climates to young athletes’ self-esteem, performance anxiety, and autonomous motivation: who is more influential? *Journal of Applied Sport Psychology*, *26*(4), 395–408. 10.1080/10413200.2014.907838.

[CR48] Owusu-Sekyere, F., & Gervis, M. (2016). In the pursuit of mental toughness: Is creating mentally tough players a disguise for emotional abuse? *International Journal of Coaching Science*, *10*(1), 3–24.

[CR49] Parent, S., Fortier, K., Vaillancourt-Morel, M.-P., Lessard, G., Goulet, C., Demers, G., Paradis, H., & Hartill, M. (2019). Development and initial factor validation of the Violence Toward Athletes Questionnaire (VTAQ) in a sample of young athletes. *Loisir et Société/Society and Leisure*, *42*(3), 471–486. 10.1080/07053436.2019.1682262.

[CR50] Pinheiro, M. C., Pimenta, N., Resende, R., & Malcolm, D. (2014). Gymnastics and child abuse: An analysis of former international Portuguese female artistic gymnasts. *Sport, Education and Society*, *19*(4), 435–450. 10.1080/13573322.2012.679730.

[CR51] Quested, E., & Duda, J. (2011). Perceived autonomy support, motivation regulations and the self-evaluative tendencies of student dancers. *Journal of Dance Medicine and Science*, *15*(1), 12. 21703088

[CR52] R Core Team (2020). *R: A language and environment for statistical computing*. R Foundation for Statistical Computing. https://www.R-project.org/

[CR53] Reinboth, M., Duda, J., & Ntoumanis, N. (2004). Dimensions of coaching behavior, need satisfaction, and the psychological and physical welfare of young athletes. *Motivation and Emotion*, *28*(3), 297–313. 10.1023/B:MOEM.0000040156.81924.b8.

[CR54] Revelle, W. (2021). psych: Procedures for Psychological, Psychometric, and Personality Research. Northwestern University. Retrieved on July 15, 2021 from https://CRAN.R-project.org/package=psych. Accessed 15 Jul 2021

[CR55] Ruiz, M. C., Appleton, P. R., Duda, J., Bortoli, L., & Robazza, C. (2021). Social environmental antecedents of athletes’ emotions. *International Journal of Environmental Research and Public Health*, *18*(9), 4997. 10.3390/ijerph18094997. 34066860 10.3390/ijerph18094997 PMC8125922

[CR56] Smits, F., Jacobs, F., & Knoppers, A. (2017). ‘Everything revolves around gymnastics’: Athletes and parents make sense of elite youth sport. *Sport in Society*, *20*(1), 66–83. 10.1080/17430437.2015.1124564.

[CR57] Stebbings, J., Taylor, I. M., & Spray, C. M. (2015). The relationship between psychological well- and ill-being, and perceived autonomy supportive and controlling interpersonal styles: A longitudinal study of sport coaches. *Psychology of Sport and Exercise*, *19*, 42–49. 10.1016/j.psychsport.2015.02.002.

[CR58] Stirling, A., & Kerr, G. (2008). Defining and categorizing emotional abuse in sport. *European Journal of Sport Science*, *8*(4), 173–181. 10.1080/17461390802086281.

[CR59] Stirling, A., & Kerr, G. (2009). Abused athletes’ perceptions of the coach-athlete relationship. *Sport in Society*, *12*(2), 227–239. 10.1080/17430430802591019.

[CR60] Stirling, A., & Kerr, G. (2013). The perceived effects of elite athletes’ experiences of emotional abuse in the coach–athlete relationship. *International Journal of Sport and Exercise Psychology*, *11*(1), 87–100. 10.1080/1612197X.2013.752173.

[CR61] Unipark EFS survey (version EFS fall 2020) (2020). Questback GmbH.

[CR63] Vertommen, T., Schipper-van Veldhoven, N., Wouters, K., Kampen, J. K., Brackenridge, C. H., Rhind, D. J. A., Neels, K., & Van Den Eede, F. (2016). Interpersonal violence against children in sport in the Netherlands and Belgium. *Child Abuse & Neglect*, *51*, 223–236. 10.1016/j.chiabu.2015.10.006. 26516053 10.1016/j.chiabu.2015.10.006

[CR62] Vertommen, T., Kampen, J., Schipper-van Veldhoven, N., Uzieblo, K., & Van Den Eede, F. (2018). Severe interpersonal violence against children in sport: Associated mental health problems and quality of life in adulthood. *Child Abuse & Neglect*, *76*, 459–468. 10.1016/j.chiabu.2017.12.013. 29253798 10.1016/j.chiabu.2017.12.013

[CR64] Willson, E., Kerr, G., Stirling, A., & Buono, S. (2021). Prevalence of maltreatment among Canadian national team athletes. *Journal of Interpersonal Violence*. 10.1177/08862605211045096. 34549664 10.1177/08862605211045096 PMC9554369

[CR13] de Wit, M., Pouwer, F., Gemke, R. J. B. J., Delemarre-van de Waal, H. A., & Snoek, F. J. (2007). Validation of the WHO‑5 Well-Being Index in adolescents with type 1 diabetes. *Diabetes Care*, *30*(8), 2003–2006. 10.2337/dc07-0447. 17475940 10.2337/dc07-0447

[CR65] World Health Organization (1998). *Use of well-being measures in primary health care-the DepCare project health for all*. Vol. E60246. WHO.

[CR66] Yabe, Y., Hagiwara, Y., Sekiguchi, T., Momma, H., Tsuchiya, M., Kanazawa, K., Koide, M., Itaya, N., Yoshida, S., Sogi, Y., Yano, T., Onoki, T., Itoi, E., & Nagatomi, R. (2019). Parents’ own experience of verbal abuse is associated with their acceptance of abuse towards children from youth sports coaches. *The Tohoku Journal of Experimental Medicine*, *249*(4), 249–254. 10.1620/tjem.249.249. 31839627 10.1620/tjem.249.249

[CR67] Zeileis, A. (2004). Econometric computing with HC and HAC covariance matrix estimators. *Journal of Statistical Software*, *11*(1), 1–17. 10.18637/jss.v011.i10.

[CR68] Zeileis, A., & Hothorn, T. (2002). Diagnostic checking in regression relationships. *R News*, *2*(3), 7–10.

[CR69] Zeileis, A., Köll, S., & Graham, N. (2020). Various versatile variances: An object-oriented implementation of clustered covariances in R. *Journal of Statistical Software*, *95*(1), 1–36. 10.18637/jss.v095.i01.

